# Antigenicity and Immunogenicity of Rotavirus VP6 Protein Expressed on the Surface of *Lactococcus lactis*

**DOI:** 10.1155/2013/298598

**Published:** 2013-07-24

**Authors:** L. E. Esteban, C. F. Temprana, M. H. Argüelles, G. Glikmann, A. A. Castello

**Affiliations:** Laboratorio de Inmunología y Virología (LIV), Universidad Nacional de Quilmes, Bernal, B1876BXD Buenos Aires, Argentina

## Abstract

Group A rotaviruses are the major etiologic agents of acute gastroenteritis worldwide in children and young animals. Among its structural proteins, VP6 is the most immunogenic and is highly conserved within this group. *Lactococcus lactis* is a food-grade, Gram-positive, and nonpathogenic lactic acid bacteria that has already been explored as a mucosal delivery system of heterologous antigens. In this work, the nisin-controlled expression system was used to display the VP6 protein at the cell surface of *L. lactis*. Conditions for optimal gene expression were established by testing different nisin concentrations, cell density at induction, and incubation times after induction. Cytoplasmic and cell wall protein extracts were analyzed by Western blot and surface expression was confirmed by flow cytometry. Both analysis provided evidence that VP6 was efficiently expressed and displayed on the cell surface of *L. lactis*. Furthermore, the humoral response of mice immunized with recombinant *L. lactis* was evaluated and the displayed recombinant VP6 protein proved to be immunogenic. In conclusion, this is the first report of displaying VP6 protein on the surface of *L. lactis* to induce a specific immune response against rotavirus. These results provide the basis for further evaluation of this VP6-displaying *L. lactis* as a mucosal delivery vector in a mouse model of rotavirus infection.

## 1. Introduction

*L. lactis* is a food-grade, Gram-positive, and nonpathogenic lactic acid bacteria that has already been explored as a mucosal delivery system of heterologous antigens [1]. The most commonly used system for protein expression in *L. lactis* is the nisin-controlled expression system (NICE) based in the combination of a vector containing a nisin-inducible promoter (P_nisA_) and the regulatory genes (nisK and nisR) inserted in the bacterial genome of *L. lactis* strain NZ9000 [2]. Diverse genetic constructs can be used to target the heterologous antigen to different cell compartments (cytoplasm, cell wall, or extracellular medium). In particular, for cell-wall anchoring, the antigen can be fused to a fragment of *Streptococcus pyogenes* M6 protein which allows peptidoglycan binding of the heterologous protein [3, 4].

Rotavirus particles are nonenveloped with a triple-layered protein capsid, belonging to the *Reoviridae* family. Among them, group A rotaviruses are the major etiologic agents of acute gastroenteritis worldwide in children and young animals. The rotavirus diarrhea is associated with a high mortality rate, particularly in developing countries, as well as to a considerable economic burden. These facts have led to an extensive research in rotavirus vaccinology to prevent its morbidity and mortality [5–7].

Rotavirus protein expression in *L. lactis* was previously reported by Perez et al. [8] (VP7 protein), Marelli et al. [9], Rodríguez-Díaz et al. [10] (VP8* protein), Li et al. [11] (VP4 protein), and Enouf et al. (NSP4 protein) [12]. However, among rotavirus structural proteins, the intermediate layer protein VP6 is the most immunogenic and determines group specificity since it is highly conserved among strains belonging to the same group [13]. When coadministered with an adjuvant and tested in the murine infection model, VP6 (as the only viral antigen) induced a protective immune response. This protection did not depend on the murine rotavirus strains used for the challenge, revealing that VP6 contains at least some of the epitopes shared between strains. Moreover, the fact that crossed protection was induced by two divergent VP6 proteins indicates that a vaccine including VP6 from any group A rotavirus would protect against infection with any other group A rotavirus strain [14, 15].

In the present paper, *L. lactis*  NZ9000 was evaluated as a cell-wall display vector of rotavirus VP6 protein and used as an antigenic carrier for mice immunization.

## 2. Materials and Methods

### 2.1. Bacterial Strains and DNA Manipulation

*L. lactis* strain NZ9000, kindly provided by Dr. Christian Magni (Instituto de Biología Molecular y Celular de Rosario, Argentina), was grown in M17 broth (Biokar Diagnostics, Beauvais, France) supplemented with 0.5% glucose at 30°C without shaking. *Escherichia coli* strain TOP10 was grown in the Luria-Bertani medium at 37°C with shaking. Clones were selected by the addition of antibiotics as follows: for *L. lactis*, chloramphenicol 10 *µ*g/mL and for *E. coli*, ampicillin 100 *µ*g/mL. 

Plasmid DNA isolation and general procedures for DNA manipulations were essentially performed as described previously [16]. Reverse transcriptase reaction was performed using AMV RT (Promega, Madison, WI, USA) polymerase chain reactions (PTC-200 Thermo Cycler, MJ Research, Waltham, MA, USA) were performed using Pfu DNA polymerase or Taq DNA polymerase (Promega, Madison, WI, USA). Plasmids were sent to Macrogen Sequencing Service (Seoul, Republic of Korea) for DNA sequencing.

### 2.2. Viruses and Cell Culture

EC rotavirus strain was used to generate VP6-encoding cDNA, while RRV rotavirus strain was used to produce a virus stock providing antigens for ELISA and Western blot assays. The EC strain of rotavirus was kindly provided by Dr. Harry Greenberg (Department of Medicine and Microbiology and Immunology, Stanford University School of Medicine, Stanford, CA, USA) and RRV strain was kindly provided by Dr. Viviana Parreño (Instituto de Virología, CICVyA, INTA Castelar, Argentina). Both strains were grown in confluent MA104 cells maintained in Dulbecco's modified Eagle's medium without serum and with 2 *µ*g/mL trypsin. To be used as antigen for ELISA and Western blot assay, cell-culture-propagated RRV strain was concentrated by ultracentrifugation through a sucrose cushion and further purified on a cesium chloride gradient as previously described [17].

### 2.3. Construction of VP6 Expression Plasmid

EC rotavirus strain VP6 cDNA was obtained by a reverse transcriptase reaction/polymerase chain reaction (RT/PCR) procedure from viral RNA extracted as previously described [18]. The primers' sequences were 5′ ATGGATGTGCTGTACTCC 3′ and 5′ CTTTACCAGCATGCTTCTA 3′. The PCR product was cloned into pGEM-T Easy Vector (Promega, Madison, WI, USA) and the resulting plasmid pGEM-VP6 was transformed into *E. coli* strain TOP10 cells.

To target VP6 to the surface of *L. lactis*, the plasmid pCWA:Nuc, kindly provided by Dr. Christian Magni (Instituto de Biología Molecular y Celular de Rosario, Argentina) [3, 19], was used to clone the VP6 sequence under the transcriptional control of P_nisA_, in frame with the signal peptide SP (from *L. lactis* MG1363 Usp45 protein) and the cell wall anchor CWA (fragment from *S. pyogenes *M6 protein). For this, VP6 from pGEM-VP6 was PCR amplified with primers: 5′ CCAATGCATCAATGGATGTGCTGTACTCC 3′ and 5′ CCGATATCCCCTTTACCAGCATGCTTCTA 3′ containing NsiI and EcoRV restriction sites (underlined), respectively. Both the PCR product (1212 bp) and the plasmid pCWA:Nuc were digested with the same enzymes, ligated to obtain pCWA:VP6 plasmid, and transformed into *L. lactis *strain NZ9000 cells (NZ9000/pCWA:VP6) [20].

### 2.4. Conditions for Nisin Induction

To evaluate the effect of different nisin concentrations on bacterial growth and protein expression, overnight cultures of *L. lactis* NZ9000/pCWA:VP6 were used to inoculate fresh medium at a 1 : 20 dilution. After reaching different optical densities at 600 nm (OD_600_) (0.2, 0.5, and 0.8), cultures were induced with nisin (Danisco, Grindsted, Denmark) at different concentrations (0, 1, 10, 50, 100, 200, and 500 ng/mL) and cytoplasmic and cell wall protein extracts were prepared as indicated below every hour until 6 h after induction [20]. Overnight incubation was also evaluated.

### 2.5. Protein Expression and Localization Analysis

Cell wall and protoplast fractionation as well as protein extractions were performed as previously described [20]. Briefly, a volume of bacterial culture corresponding to 3 OD_600_ was centrifuged for 3 min at 10,000 g. The cell pellet was washed once with TES buffer (10 mM Tris-HCl pH 5.8, 1 mM EDTA, 25% sucrose) and then, the bacterial cell walls were digested with 200 *µ*L of TES-LLP (TES buffer containing 10 mg/mL lysozyme, 100 *µ*g/mL lysostaphin, and 1 mM phenylmethylsulfonyl fluoride). After 1 h of incubation at 37°C, the protoplasts were recovered by a 10 min centrifugation at 2,000 g. The pellet was washed with TES buffer and resuspended in 100 *µ*L of TES buffer : PAGE loading buffer (1 : 1). The supernatant (cell wall fraction) was directly mixed with 50 *µ*L of loading buffer for PAGE analysis. To obtain total protein extracts, 50 *µ*L of SDS (20%) was added after cell wall digestion and a 1 : 1 dilution was made with PAGE loading buffer.

Bacterial protein extracts were subjected to SDS-PAGE and Western blot analysis. For this, SDS-PAGE (10%) gels were Coomassie blue-stained or blotted onto PVDF membranes. After blocking with PBST (PBS, 0.2% Tween-20) containing 1% casein at 4°C overnight, the membranes were incubated with a 1/3000 dilution of a mouse polyclonal antibody anti-RRV rotavirus at 37°C for 1 h. Then, the membranes were washed and incubated with a 1/1000 dilution of HRP-conjugated goat anti-mouse IgG (Pierce Biotechnology, Rockford, IL, USA) at 37°C for 1 h followed by detection with a chemiluminescent substrate (PBL, Bernal, Argentina) according to the manufacturer's instructions. Purified and concentrated RRV rotavirus proteins were included as a positive control.

To further confirm the display of the VP6-CWA fusion protein on the bacterial surface, induced *L. lactis* NZ9000/pCWA:VP6 cultures were analyzed by flow cytometry. For this, cells were centrifuged and washed with PBS and incubated with an anti-VP6 mouse monoclonal antibody diluted in PBST containing 1% casein for 30 min at 37°C. This antibody was produced according to standard procedures [21] by fusing myeloma cells with splenocytes obtained from mice immunized with purified rotavirus. After washing, cells were incubated with a FITC-conjugated rabbit anti-mouse IgG (Caltag Laboratories, Burlingame, CA, USA). For each sample, 100,000 events were acquired on a FACScalibur flow cytometer (Becton Dickinson, Immunocytometry Systems, San Jose, CA, USA) by gating *L. lactis *on forward scatter and side scatter dot plots. A band-pass filter of 530 nm (515 to 545 nm) was used to register the cells emitting green fluorescence (FL1-H). Uninduced *L. lactis* NZ9000/pCWA:VP6 cultures were processed and stained in the same way and used as negative control.

### 2.6. Preparation of Bacterial Cells for Immunization

Bacterial cultures were optimally induced and cell pellets were washed three times with PBS. Induced (NZ9000/pCWA:VP6) or plasmid-free bacteria (NZ9000) were resuspended in PBS to obtain 10^10^ colony-forming units/mL (CFU/mL). 

### 2.7. Mice Immunization and Sample Collection

Groups of ten male BALB/c mice (6 to 8 weeks old) were inoculated subcutaneously on days 1, 14, and 28 with 1 × 10^9^ CFU of induced *L. lactis *NZ9000/pCWA:VP6. Control groups of mice received identical quantities of plasmid-free bacteria NZ9000 or PBS. The final dose volume per mouse was 100 *µ*L. Mice were bled on days 0, 14, 28, and 43 and sera were stored individually at −20°C until use. All animal procedures were conducted in accordance with the regulations of the Ethics Committee of the Universidad Nacional de Quilmes.

### 2.8. ELISA Analysis of Mice Sera

ELISA 96-well plates were coated overnight at 4°C with concentrated RRV rotavirus strain in carbonate buffer. Sera were tested in twofold dilution series (in PBST containing 1% casein) and plates were incubated for 1 h at 37°C. Mouse polyclonal antibodies anti-RRV rotavirus was used 1/400,000 as a positive control. HRP-conjugated goat anti-mouse IgG (Fc) antibodies (Pierce Biotechnology, Rockford, IL, USA) were added to the plates for 1 h at 37°C. Between steps, plates were washed three times with PBST. The o-phenylenediamine peroxidase substrate was then used for detection. The reaction was stopped after 15 min with sulfuric acid and the OD_490_ was determined. Titers were determined by testing twofold serial dilutions of mice sera. The last dilution that showed a positive signal corresponds to the final titer. The ELISA cut-off is equal to the mean OD_490_ of a set of 50 negative serum samples plus 3 standard deviations (OD = 0.11). ELISA titers were analyzed using the Kruskal-Wallis nonparametric test, with Dunn's post test for differences between groups.

### 2.9. Western Blot Analysis of Mice Sera

Purified and concentrated RRV rotavirus proteins were separated by SDS-PAGE (10%) and transferred onto PVDF membranes. After blocking with PBST containing 1% casein, the membranes were incubated with mice serum samples (1/100 dilution). Mouse polyclonal antibodies anti-RRV rotavirus were used 1/1000 as positive control. After washing, membranes were incubated with HRP-conjugated goat anti-mouse IgG (Pierce Biotechnology, Rockford, IL, USA) followed by detection with a chemiluminescent substrate (Kalium Technologies, Bernal, Argentina).

## 3. Results and Discussion

### 3.1. Expression of VP6-CWA in *L. lactis*

**Figure 1:**
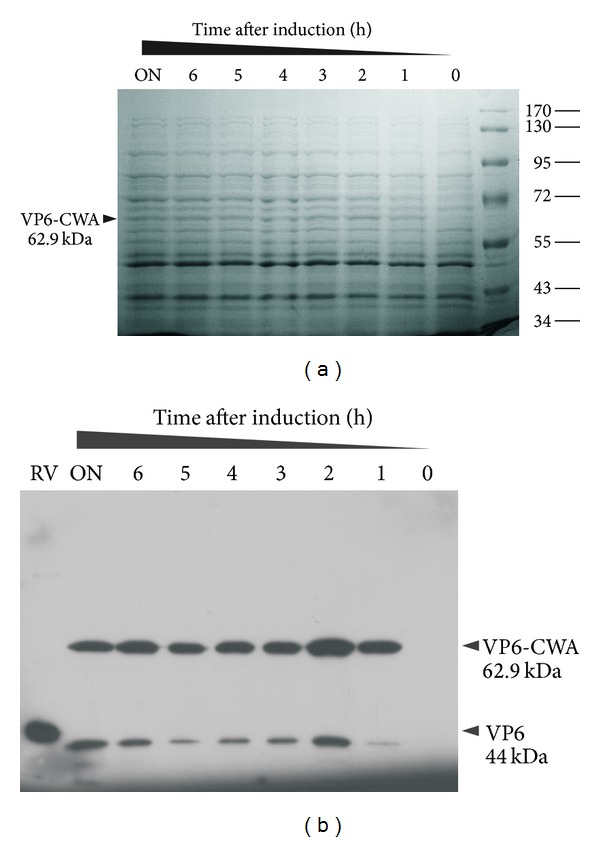
Expression analysis of VP6-CWA. (a) Coomasie-blue-stained SDS-PAGE of *L. lactis* NZ9000/pCWA:VP6 total protein extracts at different times after induction. (b) Western blot analysis of induced *L. lactis* NZ9000/pCWA:VP6 total protein extracts at different times after induction to detect VP6 with a polyclonal serum against rotavirus. Rotavirus particles protein extract was included as a positive control (RV). The sizes of molecular weight standards (MWS) (in kilodaltons) are indicated between the gel and the membrane and the expected sizes of the VP6-CWA or VP6 band are denoted on the left or right of the gel or membrane, respectively. ON: overnight incubation.

The VP6 sequence from the murine rotavirus EC strain was PCR amplified, cloned, and transferred into *L. lactis *NZ9000 obtaining NZ9000/pCWA:VP6. The nucleotide sequence analysis confirmed that there were no variations in the VP6 sequence and that it was in frame with both SP and CWA fragments. To determine if pCWA:VP6 allowed the expression of VP6-CWA, total protein extracts of induced cultures were analyzed by SDS-PAGE. As can be seen in Figure 1(a) after only one hour of induction, one band corresponding to the expected size of the VP6-CWA fusion (62.9 kDa) was detected. It is important to note that this protein was absent in the extract from uninduced cultures (Figure 1(a), lane 0 h). The identity of this band was confirmed, since it reacted with a polyclonal serum against rotavirus used in Western blot analysis (Figure 1(b)). One major band was detected at the expected size of the VP6-CWA fusion protein. Degradation products of smaller molecular mass were also detected in induced samples, but none of them corresponded to native VP6. Moreover, no immunoreactive protein bands were found in the extract from uninduced cultures (Figure 1(b), lane 0 h). These results show that VP6 can be expressed in *L. lactis* and is undoubtedly recognized by specific antibodies against rotavirus (Figure 1(b), lane RV).

### 3.2. VP6 Expression Optimization and Localization

**Figure 2:**
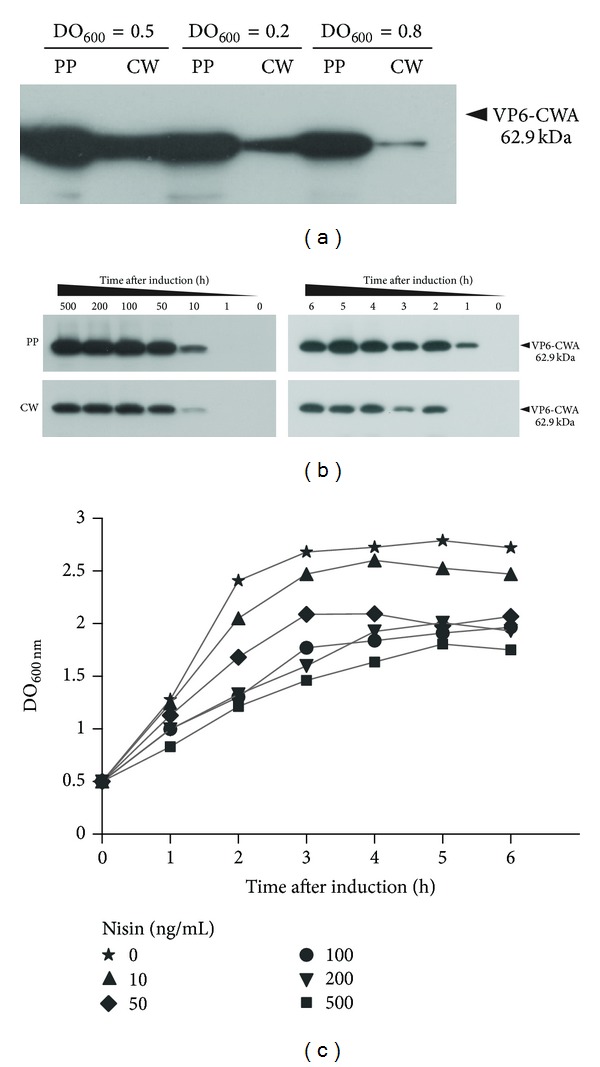
Optimization of VP6-CWA expression and cellular localization analysis. Western blot analysis of (a) protoplast (PP) and cell wall (CW) protein fractions of cultures of *L. lactis* NZ9000/pCWA:VP6 obtained two hours after induction with 100 ng/mL of nisin after reaching different OD_600_. (b) Protoplast (top blots) and cell wall (bottom blots) protein fractions of cultures of *L. lactis* NZ9000/pCWA:VP6 induced at 0.5 OD_600_ for 2 h with increasing concentrations of nisin (left blots) and induced at 0.5 OD_600_ with 100 ng/mL of nisin at different time points (right blots). (c) Graph showing OD_600_ at different time points after induction, to analyze the effect of different nisin concentrations (in ng/mL) on the growth of *L. lactis* NZ9000/pCWA:VP6.

To confirm that VP6-CWA fusion protein was properly attached to the cell wall, cultures of *L. lactis* NZ9000/pCWA:VP6 were analyzed by cell fractioning and Western blot of protein extracts using an antirotavirus polyclonal serum. Analysis of the protein content of the cell wall fraction (CW) revealed the presence of the band corresponding to VP6-CWA, which as expected was also detected in the protoplast fraction (PP) [19] (Figure 2(a)). A multiple-banding pattern was observed in both fractions (data not shown) as seen in total cell extracts (Figure 1(b)). VP6 was not detected in the supernatant fraction of induced cultures, even after concentrating with trichloroacetic acid (data not shown) [20]. 

To determine optimal expression conditions, cultures were fractioned and analyzed by Western blot at different time points between 0 and 6 h after induction at different starting OD_600_ (0.2, 0.5, and 0.8) and increasing nisin concentrations (0, 1, 10, 50, 100, 200, and 500 ng/mL). Figures 2(a) and 2(b) show example blots obtained during expression optimization and Figure 2(c) shows growth curves plotted for every nisin concentration. To summarize, VP6-CWA expression was found to be optimal when *L. lactis* NZ9000/pCWA:VP6 cultures were induced at 0.5 OD_600_ for two hours at 30°C with 100 ng/mL of nisin, which showed the highest targeting efficiency with minimal bacterial growth impact. These conditions were used for further experiments.

The targeting efficiency of VP6-CWA (the ratio of VP6-CWA protein detected in the cell wall as a fraction of total VP6-CWA protein detected) could be estimated to be around 40% under optimal conditions as determined by Western blot densitometry. The band considered for this estimate was that corresponding to undegraded VP6-CWA. This means that VP6-CWA is efficiently exported to the cell wall, in accordance with previous results obtained with *L. lactis* NZ9000/pCWA:Nuc [19].

**Figure 3:**
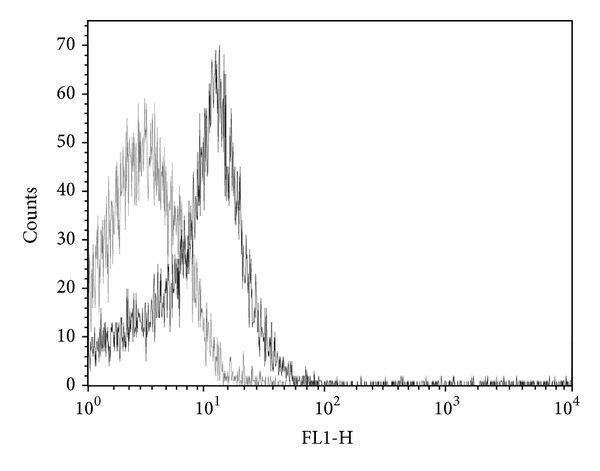
Flow cytometry analysis of VP6 surface expression. Histogram overlay of induced (black line) or uninduced (grey line) cultures of *L. lactis* NZ9000/pCWA:VP6.

In order to confirm VP6 localization, optimally induced *L. lactis* NZ9000/pCWA:VP6 cultures were analyzed by flow cytometry (Figure 3). The right shift to higher fluorescence values observed for induced *L. lactis *(Figure 3, black line) not only confirmed VP6 was present on the cell wall, but it also reflected that the protein was properly exposed on the outer side of the *L. lactis* NZ9000/pCWA:VP6 cell wall.

### 3.3. Immunogenicity of VP6-CWA in Mice

**Figure 4:**
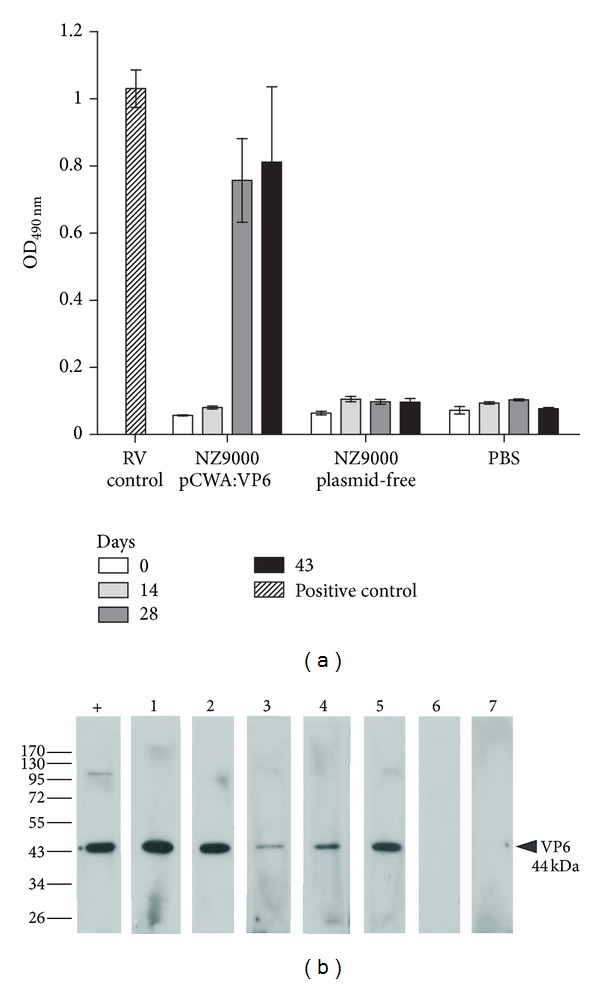
Humoral immune response in mice immunized with *L. lactis* NZ9000/pCWA:VP6, NZ9000, or PBS. (a) Anti-VP6 IgG levels in mice sera collected at days 0, 14, 28, and 43 after first inoculation as determined by ELISA. Bars represent mean OD_490_ and error bars represent SEM for each group. Mouse polyclonal antibodies anti-RRV rotavirus was used as a positive control. (b) Western blot analysis of VP6-specific IgG in mice sera collected at day 43 after first inoculation with NZ9000/pCWA:VP6 (lanes 1 to 5), NZ9000 plasmid-free (lane 6), or PBS (lane 7). Representative membranes strips individually probed with sera diluted 1/100 are shown. Mouse polyclonal antibodies anti-RRV rotavirus was used as a positive control (+). The sizes of molecular weight standards (MWS) (in kilodaltons) and the size of the VP6 band are denoted on the left and right of the membrane, respectively.

To evaluate whether VP6-CWA produced by *L. lactis* could induce a VP6 specific humoral response, 1 × 10^9^ CFU of *L. lactis* NZ9000/pCWA:VP6 were used to immunize mice on a three-dose schedule via the subcutaneous route. Immune sera were analyzed by testing their reactivity against VP6 by ELISA (Figure 4(a)) and Western blot (Figure 4(b)). Mice immunized with *L. lactis* NZ9000/pCWA:VP6 exhibited a marked increase in specific serum IgG compared to mice immunized with plasmid-free NZ9000 or PBS. Rotavirus-specific antibodies could be detected in sera at day 28 following first immunization while sera from control mice remained negative after completing the immunization protocol (Figure 4(a)). The highest anti-VP6 IgG titer was obtained after the third booster immunization reaching an average endpoint titer of 2280 (95% confidence interval: 1000–3560) for mice immunized with *L. lactis* NZ9000/pCWA:VP6 and was found to be significantly different when compared to sera from control mice immunized with *L. lactis *NZ9000 (*P* < 0.05) or PBS (*P* < 0.01). Representative membrane strips for sera from each immunized group analyzed by Western blot are shown in Figure 4(b) and indicate that sera from mice immunized with *L. lactis* NZ9000/pCWA:VP6 were directed against VP6, while sera from control mice did not recognize rotavirus proteins.

Although antigen production at the *L. lactis *cell wall is in general less efficient compared to intracellular production [22], the obtained results indicated that the amount of VP6, produced by genetically engineered *L. lactis* NZ9000/pCWA:VP6, was sufficient for eliciting a specific immune response against rotavirus. 

Moreover, it has been previously described that, for some antigens expressed in *L. lactis*, only cell-wall associated but not secreted or intracytoplasmic expression strategies were able to induce specific IgG in serum [23]. On the contrary, other antigens like tetanus toxin fragment C (TTFC) [24–26] did induce a specific immune response regardless of the subcellular localization. This demonstrates that not only the subcellular location of antigen expression affects the immune response generated, but the antigen's characteristics are relevant as well. 

In this particular case, VP6 was chosen as the rotavirus antigen for expression in *L. lactis*, considering that although antibodies against the VP6 protein are not associated with classical extracellular neutralizing activity, they have been associated with protection in some studies. For example, it has been demonstrated that monoclonal antibodies directed against VP6 protect mice against rotavirus infection by intracellular interference of the viral cycle, when hybridoma cells are injected into the backs of immunodeficient mice [27, 28]. In addition, a DNA vaccine encoding VP6 induced protective active immunity in mice [29, 30], and an *E. coli-*expressed fusion VP6-maltose-binding protein or a 14-amino-acid VP6 peptide induced protection from viral challenge [31]. Furthermore and importantly, this protection is heterotypic since VP6, which represents 51% of the virion mass, is antigenically conserved among most circulating group A rotavirus strains [14, 32, 33]. 

The results obtained so far with *L. lactis* NZ9000/pCWA:VP6 indicate that the VP6 protein is efficiently expressed and correctly displayed on the cell wall and that specific antibodies can be elicited, demonstrating correct folding of the epitopes and good immunogenicity. These recombinant bacteria can now be further explored as a mucosal delivery vehicle to be administered via oral or intranasal routes. In this sense, mucosal immunization is mandatory to determine if a protective immune response against rotavirus can be elicited, by testing this VP6-expressing vector on the mouse model of rotavirus infection [34–36]. Furthermore, research on inoculation routes as well as immunization protocols will bring insight into this bacterial display platform strategy, suggesting a safer alternative to attenuated viral pathogens, which is the current strategy for human immunization against rotavirus [5, 7]. Additionally, these bacterial vectors expressing heterologous proteins are economical to produce and have a great potential for large-scale use of the NICE system [37]. 

## 4. Conclusions

This is the first time *L. lactis *surface display system was exploited as a means of expressing the rotavirus VP6 protein in the form of an immunogenic cell-wall anchored fusion protein. VP6 expression levels were optimized in order to improve cell-wall anchoring and surface exposure. Furthermore, *L. lactis *NZ9000/pCWA:VP6 proved to induce rotavirus-specific serum antibodies in a mouse model in the absence of any exogenous adjuvant. These results provide the basis for this bacterial vector to be further evaluated as a strategy for mucosal immunization against rotavirus in a mouse model of rotavirus infection.
